# An Experimental Inquiry into the Effects of Tonics, and Other Medicinal Substances on the Cohesion of the Animal Fibre

**Published:** 1817-05

**Authors:** 


*An experimental Inquiry into the Effects of Tonics, and other Medicinal Substances on the Cohesion of the Animal Fibre.* By the late ADAIR CRAWFORD, M. D. 8vo. pp. 124, plates, London, 1816.

THESE experiments were prepared for the press upwards of twenty years ago, and are now published by Dr. Crawford of Lisburne, the author's brother. They certainly bear the marks of industry, ingenuity, and just reasoning. The latter indeed might have undergone some modification, had the author lived to see the revolution which chemistry has experienced; but the *facts* remain, and the remarkable changes produced on the animal fibre by the application of so many different substances must excite the attention, and stimulate the industry of others to prosecute the investigation.

The work, as a series of experiments, is, in its nature, incapable of analysis, and therefore we can only offer a few specimens of the facts and reasonings contained therein, referring to the work itself for a careful perusal.

*Effects of vinous and spirituous Liquors.*—From experiments on the intestines of kittens it appears that firmness, elasticity, and strength are considerably increased by immersion in port wine—but these experiments were on the dead intestine; and therefore of not so much value. The effects of sherry wine were still greater. Dr. Crawford thinks that the same effects, somewhat modified, are probably produced on the living fibre. The following experiments were made with that view. To two kittens of the same litter he gave, to one port wine and milk; to the other, milk alone. In one hour they were drowned. The stomach of the kitten that had taken the wine was capable of bearing a greater weight before it tore than the other, and was much firmer to the touch. "It contained less gastric juice in a separate state, and it had acquired a faint red colour." It is curious that the cohesion of the *skin* of these animals was greatly *diminished* by immersion in wine. In respect to the effects of spirits on the intestinal fibre, it appeared "that the firmness, elasticity, and strength of the small intestines of a kitten were increased by immersing them for a short time in rum." p. 20.

Two kittens were selected from the same litter. One was drowned, and the other was killed by giving it a table spoonful of rum. In this instance the cohesions of their stomachs were very nearly equal; but the stomach of the kitten which had swallowed the rum was very much inflamed.

From these experiments Dr. C. draws some pathological conclusions, which are more than doubtful.

"As wine," says he, "augments the tone and vigour of the stomach, and at the same time relaxes and debilitates the skin, does it not by these properties determine from the centre to the surface of the body? And hence, does it not, when judiciously administered, assist in preventing morbid affections from falling upon parts which are essential to life, and conspire with the efforts of Nature in throwing off what is injurious to the pores of the skin?" p. 24.

We fear that in a pathological point of view this theory is erroneous, and in a practical, dangerous. In the tepid bath and antimonials we have the means of determining to the surface, without danger of increasing generally the force of the circulation, which wine must do; and for preventing morbid affections from falling upon vital organs, surely venæsection and other evacuations are preferable means to the stimulating operations of wine.

Some ingenious remarks, and much theoretical reasoning follow on the effects of narcotics, particularly opium; but we cannot condense them. From Dr. Crawford's experiments, however, it appears that opium increases the cohesion of the intestines, and relaxes and debilitates the skin and nerves. Astringent vegetable substances, as galls, Peruvian bark, &c. were found to strengthen the dead animal fibre, as might *a priori* be expected; but it was somewhat curious, that the elasticity and strength of the skin were diminished thereby. We shall here introduce a passage relative to the effects of salt.

"From the preceding experiments it appears, that common salt greatly increases the cohesion of all the soft parts of the animal body; hence, probably, the reason why this salt, which is furnished in such abundance to the inhabitants of the earth, is so admirably calculated to preserve, and, in many cases, to restore health. We have seen in particular that it possesses the power of augmenting, in an extraordinary degree, the tone and strength of the arterial system. And as during life the force which must be incessantly exerted by the heart and arteries to communicate motion to the sanguineous mass is very great; it is obvious, that if this force be diminished by any of the debilitating causes to which animal life is incident, the general health will be impaired.

"Common salt is excellently adapted to counteract the influence of such causes; for it not only acts as a general corroborant, but it is peculiarly calculated to support the tone and strength of that part of the animal body in which the greatest effort is required." P. 77.

Sulphate of magnesia was likewise found to increase the strength and elasticity of the skin, the blood-vessels, nerves, and intestines.

"Hence," says our author, "probably, the reason why this salt in small doses, frequently repeated, is one of the most efficacious remedies hitherto discovered in constipations arising from inflammation in the bowels."

Oxymuriate of mercury greatly increased the elasticity and strength of the intestines, skin, blood-vessels, and nerves of animals deprived of life.

### CONCLUSION.

"It is a well known law," says Dr. C. "of the animal œconomy, that when changes are produced in certain parts of the body, there are other parts which undergo corresponding changes. The sympathetic motions which thus take place, have been traced with great care by physiologists, and various theories have been formed to account for them.

"It appears to follow from the preceding experiments, that some parts of the body are so constituted, as to be subject to a different law, which has hitherto been but slightly noticed by physiologists. Thus, from the opposite effects produced upon the intestines and skin, by the same medicinal substances, it would seem that a change in the one is frequently associated with a contrary change in the other.

"There are many facts ascertained by the observation of practical physicians, which appear to confirm this law of association. It has been observed, for example, by Sydenham, and by other writers, that in the beginning of fevers, warm clothing and tepid fomentations applied to the skin, will frequently remove vomiting and purging. In this case, it is probable, that at the commencement of the disease, the skin is constricted and the stomach relaxed; and, that by taking off the constriction from the former, the latter, in some measure, recovers its tone. In like manner, a blister applied to the back, by relaxing the skin, will communicate tone to the stomach, and will relieve dyspepsia. On the contrary, a draught of cold water, by imparting tone to the stomach, will frequently relax the skin and excite perspiration. It is probably owing to the same law of association, that vomiting and purging are often produced by the application of cold air to the skin in the autumnal season.

"Dr. Cullen has shewn, that a constriction of the skin takes place at the commencement of the paroxysm of intermittent fevers. There is, as I before observed, great reason to believe, that this constriction is accompanied with a diminution of tone in the stomach. For not to mention the facts above stated, which show that the changes in the stomach and skin frequently alternate with each other, the nausea and vomiting which usually take place upon the accession of the cold fit, render it extremely probable that the tone of the stomach is reduced below the standard of health. These symptoms are alleviated as soon as the skin begins to be relaxed.

"Hence, we may perceive the reason why substances which relax the stomach, as volatile alkali, and aromatic oils, given during the cold fit, in large doses, increase the violence of the paroxysm. But the substances on the contrary, which strengthen the stomach, and relax the skin, as emetic tartar, ipecacuanha, and opium, when exhibited at the accession of the paroxysm generally diminish its violence, and sometimes entirely remove it.

"If the state of the skin and intestines at the beginning of the fit of an intermittent be such as we have now described, it will, in some measure, explain the mode of the operation of the Peruvian bark in this class of diseases; for that medicine possesses, in a very high degree, the power of producing changes in the stomach and skin, directly opposite to those which probably take place at the commencement of the paroxysm.

"It seems to be an object of great moment to investigate the extent of the abovementioned law of association; and to ascertain, by experiment and observation, how far the changes which may arise in the several parts of the body are calculated to produce similar or dissimilar changes in other parts. The complete elucidation of this subject must be reserved for future inquiry. I shall only remark at present, that I have reason to believe, from experiment, that the changes in the skin are frequently associated with opposite changes in the lungs, as well as in the stomach and intestines." p. 124.

This doctrine of sympathy has been carried since Dr. Crawford's time much farther; and certainly is deserving of great attention in the pathology of diseases. Upon the whole, though we must object to many of Dr. Crawford's reasonings, yet his facts are interesting, and worthy of perusal.

